# Incident Cardiovascular Disease in Women With Type 1 or Type 2 Diabetes Following a Hypertensive Disorder of Pregnancy

**DOI:** 10.1161/HYPERTENSIONAHA.123.22081

**Published:** 2024-02-22

**Authors:** Kristina Mattsson, Mats Pihlsgård, Sofia Enhörning, Simon Timpka

**Affiliations:** 1 Perinatal and Cardiovascular Epidemiology, Clinical Sciences Malmö, Lund University, Sweden (K.M., M.P., S.E., S.T.).; 2 Departments of Obstetrics and Gynecology (K.M., S.T.), Skåne University Hospital, Lund and Malmö, Sweden.; 3 Internal Medicine (S.E.), Skåne University Hospital, Lund and Malmö, Sweden.

**Keywords:** eclampsia, epidemiology, gestational hypertension, preeclampsia, prevention

## Abstract

**BACKGROUND:**

The extent to which a history of hypertensive disorders of pregnancy is associated with incident cardiovascular disease also among women with diabetes is unknown.

**METHODS:**

In this nationwide register-based cohort study, parous women aged 18 to 69 years with a first delivery in the Swedish Medical Birth Register, regardless of diabetic status at that time, and a subsequent clinical visit in the Swedish National Diabetes Register were included. Time to first cardiovascular disease event (myocardial infarction, stroke, or heart failure) before age 70 years by hypertensive disorders of pregnancy history was separately analyzed by diabetes type using Cox regression models that included conventional risk factors.

**RESULTS:**

In total, 1748 (18.9%) of 9230 women with type 1 and 5904 (10.6%) of 55 773 women with type 2 diabetes had their first delivery complicated by a hypertensive disorder of pregnancy. Median time (25–75th percentile) between first delivery and start of follow-up was 3.3 (1.4–13.0) years for women with type 1 and 29.8 (22.4–35.6) years for women with type 2 diabetes. In modeling, the risk for any cardiovascular disease event among women with a history of hypertensive disorders of pregnancy was generally 10% to 20% higher, with main models estimating hazard ratios to 1.20 (95% CI, 0.99–1.47) for women with type 1 and 1.15 (95% CI, 1.02–1.29) for women with type 2 diabetes.

**CONCLUSIONS:**

In women with diabetes, a history of hypertensive disorders of pregnancy was associated with an increased risk of incident cardiovascular disease and should be considered as a risk enhancer.

**NOVELTY AND RELEVANCE**

**What Is New?**

Women with diabetes and a history of hypertensive disorders of pregnancy at their first delivery have 10% to 20% higher risk of cardiovascular disease, compared with women with diabetes but no history of hypertensive disorders of pregnancy.
A history of hypertensive disorders of pregnancy was not associated with all-cause mortality in neither patients with type 1 nor type 2 diabetes.

**What Is Relevant?**

The extent to which a history of hypertensive disorders of pregnancy is associated with incident cardiovascular disease also among women with diagnosed diabetes is unknown.

**Clinical/Pathophysiological Implications?**

A history of hypertensive disorders of pregnancy should be considered as a cardiovascular disease risk enhancer among patients with diabetes.

Despite considerable advances in the clinical management and treatment of diabetes over the last decades, patients with diabetes are at increased risk of cardiovascular disease, and face excess mortality compared with the general population.^1–4^ Compared with male patients with diabetes, women with diabetes have an excess relative risk for cardiovascular disease, regardless of diabetes type.^5–9^ The reasons for this are not fully understood; a variety of physiological, hormonal, and behavioral factors have been discussed, including disparities in the management of cardiovascular risk factors to the detriment of women.^4,10–12^ Although parous women with a history of hypertensive disorders of pregnancy (including preeclampsia) have more advanced coronary artery atherosclerosis in middle age, higher risk of developing chronic hypertension, and a higher risk of incident cardiovascular disease,^13–15^ past pregnancy history have hitherto been overlooked in the clinical care of women with diagnosed diabetes.

Clinical diabetes management aims at minimizing diabetes-related complications in the short and long term, by optimization of certain risk factors: HbA1c (glycated hemoglobin) levels, LDL (low-density lipoprotein) cholesterol levels, albuminuria, smoking, and blood pressure. Keeping these risk factors within target ranges has documented beneficial effects in terms of reducing the risk for cardiovascular events and mortality, shown for both type 1 and type 2 diabetes.^3,16^ However, it is unknown the extent to which a history of hypertensive disorders of pregnancy could help identify women with diabetes at higher risk of cardiovascular disease and whether any such risk increase differ by clinical risk factor control. Currently, cardiovascular disease prevention guidelines list pregnancy-associated conditions, such as preeclampsia as a cardiovascular disease risk-enhancing factor in primary prevention^17^ but these conditions are not referred to in the recommendations for patients with diabetes.^17–19^

In light of this research gap, our primary aim was to analyze risk of cardiovascular disease and mortality by history of hypertensive disorders of pregnancy in women with type 1 and type 2 diabetes, respectively. We sought also to examine the extent to which keeping clinical risk factors within target ranges could mitigate any risk increase.

## METHODS

### Data Availability

Because of the sensitive nature of the data collected for this study, requests to access the data from qualified researchers trained in human subject confidentiality protocols may be sent to the Swedish Board of Health and Welfare at socialstyrelsen@socialstyrelsen.se, Statistics Sweden at scb@scb.se, and the Swedish National Diabetes Registry at ndrinfo@registercentrum.se. Ethical approval was granted by the Swedish Ethical Review Authority (DNR 2019-03229).

### Study Sample

We conducted a nationwide prospective cohort study in Sweden by identifying all parous women aged 18 to 69 years with diabetes and an index visit in the Swedish National Diabetes Register 1996 to 2020. Pregnancy history pertinent to their first delivery (regardless of diabetic status at that time) was retrieved from the Swedish Medical Birth Registry. Any previous or incident cardiovascular events (myocardial infarction, stroke, heart failure) were identified through comprehensive in-hospital care and cause-of-death registers.

### Diabetes Diagnoses

Data on diabetes were retrieved from the Swedish National Diabetes Register. The register, described in more detail elsewhere,^20^ was launched in 1996 and includes almost all adult patients in Sweden with type 1 diabetes and ≈90% of patients with type 2 diabetes.^21^ The register contains information on the date of diabetes diagnosis, risk factors, diabetes-related complications, medications, and other clinical data, as well as self-reported lifestyle factors. The register does not only contain visits by patients with a set diabetes diagnosis type, why the following previously validated epidemiological criteria were used to define diabetes type: patients below 30 years of age at diagnosis with insulin treatment were classified as having type 1 diabetes, whereas type 2 diabetes was defined as having either diet treatment with or without an oral antihyperglycemic agent, or patients >40 years at first diagnosis, treated with insulin alone or in combination with antihyperglycemic agents.^2,16,20^ Patients with registrations of both type 1 and type 2 diabetes according to above were excluded.

**Figure 1.**
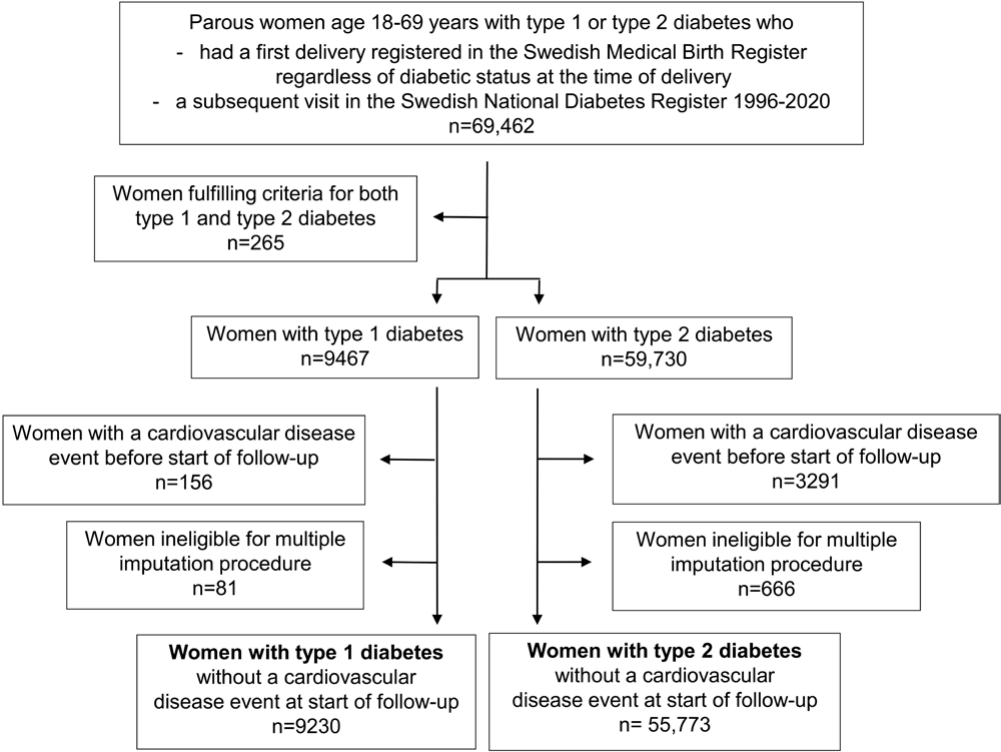
Flowchart of study samples.

Patients with any of the outcomes (myocardial infarction, stroke, heart failure, or all-cause death) registered in the Swedish In-patient Register before start of follow-up were excluded. We also excluded patients ineligible for multiple imputations as described below. Figure 1 depicts the selection process of women with type 1 and type 2 diabetes.

### Hypertensive Disorders of Pregnancy

Hypertensive disorders of pregnancy, that is, preeclampsia, eclampsia, or gestational hypertension during the woman’s first pregnancy were considered the main exposure. Data on hypertensive disorders of pregnancy were retrieved from the Swedish Medical Birth Register (which includes data on almost all deliveries in Sweden since 1973),^22^ using the Swedish version of the *International Classification of Diseases*, *Eighth to Tenth Revision* as described in the Supplemental Material.

### Covariables and Clinical Risk Factor Control

We collected data on clinical risk factors from the Swedish National Diabetes Register, including as continuous variables: systolic and diastolic blood pressure, LDL levels, HbA1c levels, and as categorical variables: smoking (yes/no), and albuminuria, where at least 2 out of 3 positive samples within 1 year was considered having albuminuria (3 categories: no or previous albuminuria, microalbuminuria, or macroalbuminuria). We also included age at study entry (continuous), and body mass index (kg/m^2^; continuous) from the same register. From Statistics Sweden we retrieved data on educational level (3 categories: primary [≤9 years], secondary [10–12 years], and postsecondary [>12 years]), country of origin (Swedish born [yes/no]), and migration.

For the analyses of number of risk factors not in control, the following cutoffs were used for defining the risk factor within the target range: ≤130/80 mm Hg for blood pressure, <53 mmol/mole for HbA1c levels, <2.6 mmol/L for LDL cholesterol, no or previous for albuminuria and lastly, no smoking at study entry. Patients were subsequently categorized as having 0–1, 2, 3, or 4–5 risk factors outside the target range.

### Cardiovascular Disease and Mortality

Data on cardiovascular events (acute myocardial infarction, stroke, or heart failure) and all-cause mortality were retrieved from the comprehensive Swedish In-patient Register (nationwide coverage of all in-patient care since 1987)^23^ and the Swedish Cause of Death Register and defined according to *International Classification of Diseases* codes as described in the Supplemental Material. In patients with type 2 diabetes, a composite cardiovascular disease outcome was analyzed, as well as separate outcomes for myocardial infarction, stroke, and heart failure, respectively. In patients with type 1 diabetes, smaller numbers only allowed analysis of the composite cardiovascular disease outcome.

### Statistical Methods

All analyses were performed separately in women with type 1 and type 2 diabetes, with women with the same diabetes type but with no history of a hypertensive disorder of pregnancy complicating their first delivery as reference group. All patients were required to have at least 1 delivery registered in the Swedish Medical Birth Register at the time of their index visit as identified in the Swedish National Diabetes Register. History of hypertensive disorders of pregnancy was defined according to first delivery irrespective of parity at this index visit. To allow for more complete data on covariables and reduce the proportion of missing data, follow-up time started 1 year after the index visit. This allowed covariable data registered at any additional clinical visit occurring during the year after the index visit to contribute to the analysis with the last entry for each variable being included. Patients who died between the index visit and start of follow-up were not included in the analyses.

Descriptive data are presented as numbers and percentages or means and standard deviations as appropriate. Crude incidence rates were calculated for cardiovascular events and all-cause mortality by hypertensive disorders of pregnancy history and are presented as events per 1000 person-years. Patients were followed until their first incident event, death (when not the outcome), migration, age 70 years, or April 1, 2021, whichever came first.

First, the study groups were compared with the log-rank tests and cumulative incidence curves. For the main analyses, we constructed Cox proportional hazard regression models for each outcome by hypertensive disorders of pregnancy history. An age-dependency in the association between the study groups and the outcomes was revealed in initial analyses and this was addressed in the Cox regression model by performing all analyses in strata of age at start of follow-up. Age was divided into 10-year intervals, starting at ages below 20 years for type 1 diabetes and below 40 years for type 2 diabetes. The proportionality of hazards was assessed graphically to check model assumptions.

We then conducted sequential adjustments: model I included age at start of follow-up as defined above. Model II included clinical risk factors: HbA1c levels, LDL levels, albuminuria, blood pressure and smoking, and model III additionally added the covariables educational level, body mass index, and whether the woman was Swedish born.

To investigate the combined risks of hypertensive disorders of pregnancy and risk factor control, 8 categories were constructed by combining a history of hypertensive disorders of pregnancy with the 4 risk factor categories as described above. Women with history of hypertensive disorders of pregnancy and the lowest risk factor level were considered the reference category. To investigate the extent to which the hypertensive disorders of pregnancy-associated risk of cardiovascular disease differed by number of risk factors, we tested the multiplicative interaction between hypertensive disorders of pregnancy history and risk factors.

Multiple imputation by chained equations was used to impute missing data on covariables for the regression analyses. To accomplish this and base imputations on a reasonable proportion of missing data, we used the most recent data on all covariables within 1 year following the index visit in as described above. The small minority of patients with missing data on more than half of all variables were excluded from all analyses. Twenty complete datasets were imputed, and the estimates from each dataset were combined using Rubin’s rule.

As outlined in the Supplemental Material, we conducted several additional analyses by varying the main analyses: using samples stratified by median age at first pregnancy and glycemic control, respectively, using hypertensive disorders of pregnancy subtypes, using history of hypertensive disorders defined according to all available deliveries, repeating the main analyses with additional covariables, and, lastly, only including women with no missing data in complete case analyses.

Estimates are presented with 95% CI. No adjustments were made for multiple testing. All analyses were performed using the statistical analysis software SAS version 9.4 (SAS Institute, Cary, NC).

## RESULTS

### Study Cohort Characteristics

In total, 9230 women with type 1 diabetes and 55 773 women with type 2 diabetes were included in the study. Median time (25–75th percentile) between first delivery and start of follow-up was 3.3 (1.4–13.0) years for women with type 1 diabetes and 29.8 (22.4–35.6) years for women with type 2 diabetes. Median main follow-up time was 13.0 years (6.5–18.8) for women with type 1 diabetes and 5.6 years (interquartile range, 2.5–9.6) for women with type 2 diabetes. Table 1 shows baseline characteristics of the study sample by diabetes type and history of hypertensive disorders of pregnancy. Women with type 2 diabetes were ≈20 years older and had higher blood pressure compared with women with type 1 diabetes irrespective of hypertensive disorders of pregnancy history. Among women with type 1 diabetes, 18.9% (n=1748) had been diagnosed with a hypertensive disorder of pregnancy during their first pregnancy; the corresponding figure for type 2 diabetes was 10.6% (n=5904).

**Table 1.**
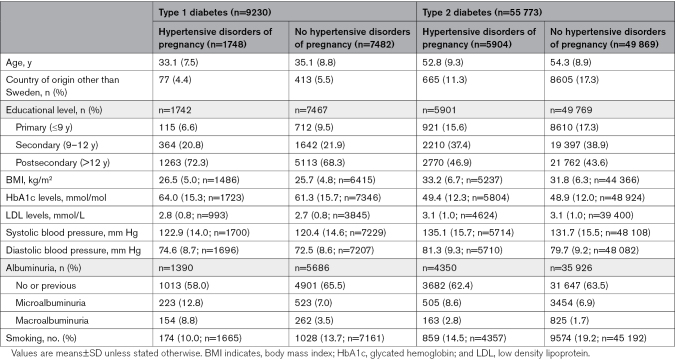
Baseline Characteristics by Type of Diabetes and History of Hypertensive Disorders of Pregnancy in First Pregnancy

### Incident Cardiovascular Disease and Mortality

**Figure 2.**
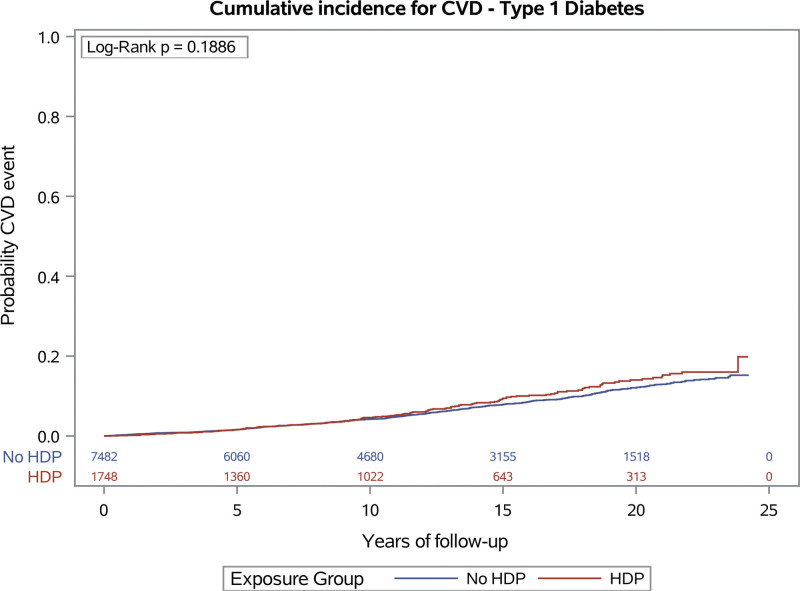
Cumulative incidence of cardiovascular disease (CVD) in women with type 1 diabetes by history of hypertensive disorder of pregnancy (HDP).

**Figure 3.**
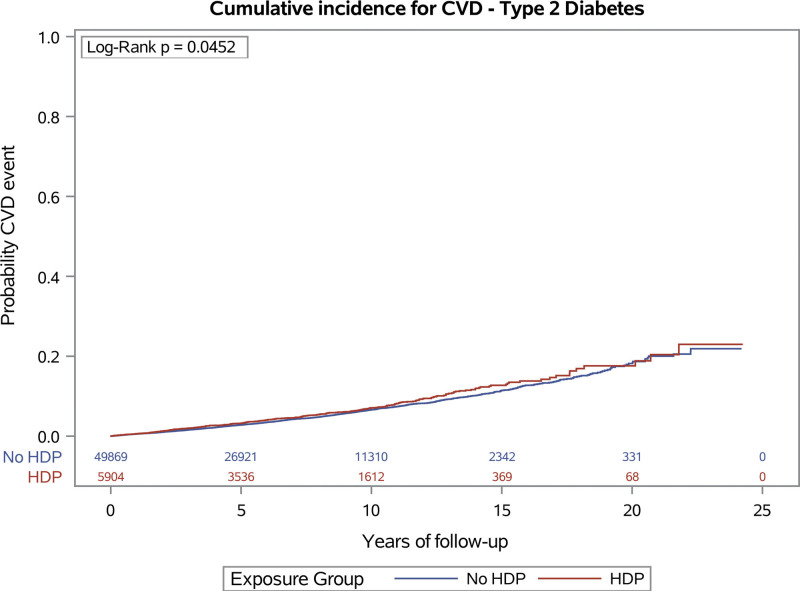
Cumulative incidence of cardiovascular disease (CVD) in women with type 2 diabetes by history of hypertensive disorders of pregnancy (HDP).

Figures 2 and 3 show the cumulative incidence rates for incident cardiovascular disease events by hypertensive disorders of pregnancy history for type 1 and type 2 diabetes, respectively.

Table 2 shows the crude incident rates and the results from modeling for women with type 1 diabetes. The risk of incident cardiovascular disease for women with a history of hypertensive disorders of pregnancy was increased by 20% in women with type 1 diabetes but there was no difference in risk of all-cause mortality.

**Table 2.**
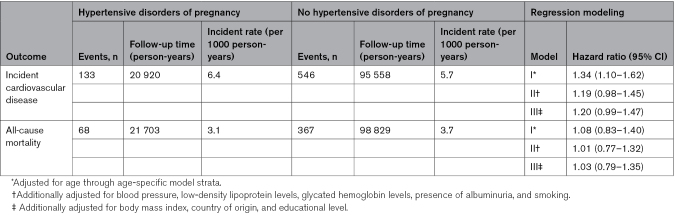
Risk of Incident Cardiovascular Disease and All-Cause Mortality in Women With Type 1 Diabetes by History of Hypertensive Disorders of Pregnancy

Table 3 shows the crude incident rates and the results from modeling for women with type 2 diabetes. The analyses showed an increased risk for incident cardiovascular disease by 15%, and the separate cardiovascular outcomes yielded similar estimates ranging from 13% to 16%. Also among women with type 2 diabetes, a history of hypertensive disorders of pregnancy was not associated with increased risk of all-cause mortality.

**Table 3.**
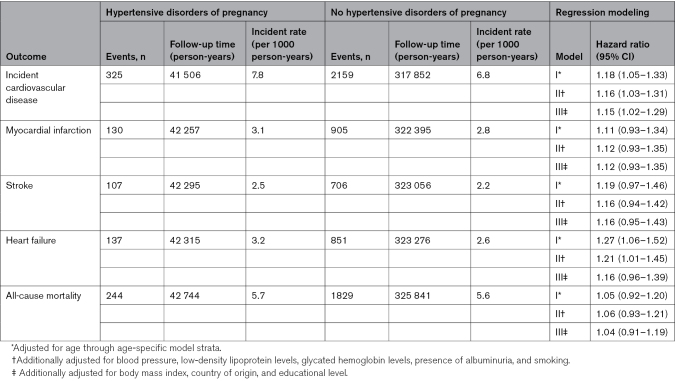
Risk of Incident Cardiovascular Disease and All-Cause Mortality in Women With Type 2 Diabetes by History of Hypertensive Disorders of Pregnancy

### Risk Factor Control

Tables S1 and S2 show hazard ratios for incident cardiovascular disease by hypertensive disorders of pregnancy history, stratified by numbers of risk factors not in control. For both diabetes types, there was a stepwise increase in risk by each level of risk factors outside target range, up to a 6-fold increase if having had both a hypertensive disorder of pregnancy and 4 to 5 risk factors outside target for type 1 diabetes and for type 2 diabetes patients. Conversely, in women with 0 to 1 risk factor, the risk of cardiovascular disease in those with a history of a hypertensive disorder of pregnancy was comparable to that in those without (Tables S1 and S2). We found no evidence of an interaction between a history of hypertensive disorders of pregnancy and number of risk factors not in control on cardiovascular disease risk (*P*=0.88 in women with type 1 diabetes and *P*=0.97 in women with type 2 diabetes).

In additional analyses (Outline Supplement), we found no evidence that main estimates varied by age at first delivery, glycemic control, or by hypertensive disorder of pregnancy subtype. Repeating the main analyses with hypertensive disorder of pregnancy history defined according to all available deliveries, or with additional covariables, had only minor effects on estimates. Forty percent (n=3728) of patients with type 1 diabetes were included in the complete case analysis (Table S3), which showed abrogated hazard ratio for incident cardiovascular disease with wide CI in model III (hazard ratio, 0.92 [95% CI, 0.60–1.43]). In contrast, among women with type 2 diabetes and no missing data (n=30 234, 54%) the hazard ratio was slightly higher (hazard ratio, 1.26 [95% CI, 1.07–1.49]) compared with the main analysis (Table S4).

## DISCUSSION

In this large nationwide register-based study, we found that women diagnosed with diabetes and a history of hypertensive disorders of pregnancy at their first delivery have a 10% to 20% higher risk for incident cardiovascular disease, compared with women with diabetes but no history of hypertensive disorders of pregnancy. Notably, this excess risk remained after consideration of conventional cardiovascular risk factors and did not differ by number of risk factors in control. As such, the risk is on top of the risk conferred by diabetes itself, and to the relatively higher cardiovascular risk of being a woman with diabetes.

Women with diabetes have been shown to fare relatively worse than men with diabetes, regardless of diabetes type. For type 2 diabetes, the excess risk for cardiovascular disease for women has been reported at 44%,^10^ with women both 15% less likely to receive recommended care, but also less likely to meet cardiovascular risk factor target levels even with treatment.^8^ The relative risk increase has been found even greater for type 1 diabetes patients: an almost tripled excess risk of coronary heart disease, ≈40% higher risk for stroke, fatal renal disease, and mortality.^5^ These sex differences have been reported consistent over all ages, but most notable for women of younger ages.^24^ In this study, a history of hypertensive disorders of pregnancy was not associated with all-cause mortality in neither in patients with type 1 nor type 2 diabetes. This might be attributable to the relatively high incident mortality observed in patients with diabetes irrespective of hypertensive disorders of pregnancy history, in combination with the only moderately (30%) increased risk of all-cause mortality in women with a history of hypertensive disorders of pregnancy previously reported.^25^ However, we did not find an increase in all-cause mortality for diabetes patients following a hypertensive disorder of pregnancy, which suggests that the increased cardiovascular disease risk is not merely a result of higher morbidity overall in these women.

In our study, we identified a subgroup of diabetic women showing an additionally increased cardiovascular risk compared with other women with diabetes, also after consideration of conventional cardiovascular risk factors. A few previous studies have investigated the joint association of hypertensive disorders of pregnancy and gestational diabetes on later cardiovascular disease risk, finding that having had both conditions confers a higher cardiovascular disease risk than having had only one of them,^26,27^ and several studies have reported a higher risk of developing a hypertensive disorder of pregnancy among patients with preexisting diabetes.^28^ The relevance of hypertensive disorders of pregnancy history for future cardiovascular health is well established,^14,29–32^ and although our results suggest that this association exists also among women with diabetes, recently published guidelines concerning cardiovascular disease prevention for patients with diabetes do not include pregnancy history at all except references to gestational diabetes.^18,19^

Some limitations of the study should be noted. Diagnostic ascertainment stems from a clinical setting, meaning that diagnostic misclassification in the registers cannot be fully avoided. However, the validity of our data sources has previously been found high,^22,23^ including the epidemiological criteria used for ascertaining diabetes type.^1–3,16,20,33^ Due to the pattern of diabetes development in the study sample, in combination with the epidemiological diagnostic criteria used, most women with type 1 diabetes had their diabetes diagnosis at the time of their first delivery whereas women with type 2 diabetes did not. Still, as the estimates for the 2 groups are similar, a history of hypertensive disorders of pregnancy is likely relevant also for women with type 1 diabetes who did not have their diabetes diagnosis at first delivery, and for women with type 2 diabetes who did. Although we could account both for several important cardiovascular risk factors and demographic confounders, there was missing data in some of the clinical risk factors. However, we used multiple imputation to address this and included data from the first year before start of follow-up. Although our analyses overall support an association between hypertensive disorders of pregnancy and incident cardiovascular disease among women with diabetes, the CIs are wide and we cannot exclude heterogeneous associations in finer strata than this study allows, such as type of diabetes further stratified by age, diabetes duration, and glycemic control. In additional analyses, the associations were also more consistent in women with type 2 diabetes than in those with type 1 diabetes. Lastly, whereas we use data from national registries, the reported associations might be different in populations where the prevalence of hypertensive disorders of pregnancy and incidence patterns of type 1 or type 2 diabetes are substantially different from those observed in Sweden.

## CONCLUSIONS

We found that a history of hypertensive disorders of pregnancy was associated with increased risk of incident cardiovascular disease in women with diabetes, but with similar risk of mortality. A history of hypertensive disorders of pregnancy should be considered as a cardiovascular disease risk enhancer among these patients given their high absolute risk of cardiovascular disease.

## PERSPECTIVES

Over the lifetime, a large majority of women deliver at least once and hypertensive disorders of pregnancy have a prevalence ≈2% to 8%. Based on the present study, these numbers are much higher among women within diabetes care (10%–20%). Few studies have investigated complications of any type by adverse pregnancy outcomes in patients with diabetes. However, a study found women with type 1 diabetes and a history of hypertensive complications during pregnancy to have increased risk of incident severe diabetic retinopathy.^34^ In addition, a history of preeclampsia has been reported to be associated with preclinical atherosclerosis in patients with type 1 diabetes.^35^ Future studies on the significance of hypertensive disorders of pregnancy history for diabetes-specific outcomes such as retinopathy and glucose control among patients with diabetes are warranted.

For diabetes patients, cardiovascular risk stratification is based primarily on age, diabetes duration, and end-organ damage, but modified through several other defined risk factors: hypertension, smoking, dyslipidemia, and obesity.^36^ Including a history of hypertensive disorders of pregnancy as a risk enhancer might help identifying women with a higher cardiovascular disease risk who otherwise would have been classified as low-risk patients, for example, relatively young patients with shorter diabetes duration. Treating these patients more intensively when appropriate could contribute to reducing the female relative excess in cardiovascular disease risk among diabetes patients.

## ARTICLE INFORMATION

### Acknowledgments

The graphical abstract was created with BioRender.com.

### Sources of Funding

The study was supported by The Swedish Research Council (2019-02082, 2022-01771), the Swedish Heart Lung Foundation (20220185), and Lund University (ALF project 2022-Projekt0112). S. Enhörning was supported by career grants from the Swedish Research Council (2022-01771) and the Swedish Society for Medical Research (SG-22-0076). No industry support was provided.

### Disclosures

S. Timpka is the local principal investigator in a multicenter study to treat preeclampsia with metformin for which Merck has provided the study drug and placebo free of charge to the sponsor. The others report no conflicts.

## Supplementary Material
