# Supplementary material for: Incident Cardiovascular Disease in Women With Type 1 or Type 2 Diabetes Following a Hypertensive Disorder of Pregnancy
Source: Hypertension. 2024 Feb 22;81(4):897–905. doi: 10.1161/HYPERTENSIONAHA.123.22081 (PMC10956670; doi:10.1161/HYPERTENSIONAHA.123.22081)
Supplement: Supplementary file 1 [file hyp-81-897-s001.docx]

**Online Supplement**

**Incident cardiovascular disease in women with type 1 or type 2 diabetes following a hypertensive disorder of pregnancy**

Mattsson et al.

**Online Methods**

**Online Results**

**Table S1.** Risk of incident cardiovascular disease in women with type 1 diabetes by history of hypertensive disorders of pregnancy and number of risk factors not in control

**Table S2.** Risk of incident cardiovascular disease in women with type 2 diabetes by history of hypertensive disorders of pregnancy and number of risk factors not in control

**Table S3.** Risk of incident cardiovascular disease in women with type 1 diabetes by history of hypertensive disorders of pregnancy in complete case analyses

**Table S4.** Risk of incident cardiovascular disease in women with type 2 diabetes by history of hypertensive disorders of pregnancy in complete case analyses

**Online Methods**

Data on hypertensive disorders of pregnancy were retrieved from the Swedish Medical Birth Register (which includes data on almost all deliveries in Sweden since 1973) using the Swedish version of the International Classification of Diseases (ICD), 8^th^-10^th^ revision. ICD-8: 637.04, 637.10, 637.01, 637.03, 637.99; ICD-9: 642D, 642E, 642F, 642G, 642H, 642X; and ICD-10: O13, O14.0, O14.1, O14.9, O15.

The following codes were used to define past and incident cardiovascular disease events, using the Swedish version of the ICD 8th-10th revisions: acute myocardial infarction (ICD-8: 410; ICD-9: 410, and ICD-10: I21), stroke (both hemorrhage and infarction) (ICD-8: 434, 436; ICD-9: 434, 436, and ICD-10: I63, I64), and heart failure (ICD-8: 428, 429, 7824; ICD-9: 428, 429X, and ICD-10: I50).

As additional analyses, we also conducted analyses in which we stratified the samples by median age at first pregnancy and glycemic control (median HbA1c), respectively. We also investigated whether there was any significant difference in association between hypertensive disorders of pregnancy subtypes (preterm preeclampsia [delivery < gestational week 37+0], term preeclampsia, and gestational hypertension) and the association with cardiovascular disease. Moreover, we repeated the main analyses with history of hypertensive disorders defined according to all available deliveries instead of only the first, i.e. all index visits then occurred after the last registered delivery. We also repeated the main analyses with additional co-variables (physical activity defined in five categories, estimated glomerular filtration rate [eGFR], and diabetes duration) with additional missing data needed to be addressed in the multiple imputation procedure as described above. Lastly, we repeated the main analyses on incident cardiovascular disease but excluded all women with any missing data.

**Online Results**

In the analyses stratified by median age at first delivery, women with type 1 diabetes ≤ median age 27 years at pregnancy had increased risk of cardiovascular disease in model III (HR 1.29 95% CI 1.00-1.66) while the estimate for those older than age 27 years did not (HR 1.13 95% CI 0.82-1.55) with p=0.22 for difference between estimates by age at first delivery. Among women with type 2 diabetes those older than median age 26 years at first pregnancy had increased risk of cardiovascular disease in model III (HR 1.24 95% CI 1.05-1.46) while those ≤ age 26 years had not (HR 1.06 95% CI 0.90-1.26) with p=0.29 for difference between estimates by age at first delivery.

In the analyses stratified by median HbA1c, women with type 1 diabetes and HbA1c ≤ median 60 mmol/mol (HR 1.12, 95% CI 0.70-1.79) had similar risk of cardiovascular disease in model III as those with HbA1c above 60 mmol/mol (HR 1.21, 95% CI 0.97-1.51, p=0.34 for difference between estimates by HbA1c level). Also among women with type 2 diabetes, women with median HbA1c below median 46 mmol/mol (HR 1.13, 95% CI 0.92-1.40) had similar risk of cardiovascular disease in model III as those above median (HR 1.16, 95% CI 1.00-1.34) with p=0.62 for difference between estimates by HbA1c level.

In neither women with type 1 diabetes (p=0.06) nor those with type 2 diabetes (p=0.12), the association with cardiovascular disease risk differed by hypertensive disorders of pregnancy subtypes. Repeating the main analyses with history of hypertensive disorders of pregnancy defined according to all available deliveries had only minor effects on the estimates in model III in both patients with type 1 diabetes (HR 1.21, 95% CI 1.01-1.45) and those with type 2 diabetes (HR 1.16, 95% CI 1.04-1.28).

When physical activity, diabetes duration, and eGFR were imputed and additionally added to model III, the association between a history of hypertensive disorders of pregnancy and cardiovascular disease was slightly attenuated (HR 1.12, 95% CI 0.92-1.37) in women with type 1 diabetes. Similarly in women with type 2 diabetes, the association between a history of hypertensive disorders of pregnancy and cardiovascular disease was also somewhat attenuated (HR 1.13, 95% CI 1.00-1.27). Among the three co-variables (physical activity, diabetes duration, and eGFR) added in this analyses, physical activity has the highest proportion of missing data (56% missing for patients with type 1 diabetes and 21% missing for those with type 2 diabetes).

| **Table S1.** Risk of incident cardiovascular disease in women with type 1 diabetes by history of hypertensive disorders of pregnancy and number of risk factors not in control | | | | |
| --- | --- | --- | --- | --- |
|  | **Crude data** (20 imputations) | | **Hazard ratios (95% CIs) from adjusted regression model**†‡ | |
|  | **Hypertensive disorders of pregnancy** | **No hypertensive disorders of pregnancy** | **Hypertensive disorders of pregnancy** | **No hypertensive disorders of pregnancy** |
| No. of risk factors not in control* | Incident rate (per 1000 person-years)† | Incident rate (per 1000 person-years)† |  |  |
| 0-1 | 2.2 | 2.1 | ref | 0.85 (0.44-1.62) |
| 2 | 5.6 | 4.8 | 2.14 (1.02–4.52) | 1.53 (0.82-2.88) |
| 3 | 8.7 | 9.9 | 3.10 (1.54-6.25) | 2.62 (1.40-4.90) |
| 4-5 | 18.4 | 20.5 | 5.92 (2.85-12.30) | 4.79 (2.59-8.85) |
| * Total number of the following risk factors not at target: blood pressure (≤130/80 mmHg), low-density lipoprotein levels (<2.6 mmol/l), glycated hemoglobin levels (< 53 mmol/mole), micro- or macroalbuminuria, and smoking, regardless of combination.  † Average incidence rate per strata across all imputed datasets.  ‡ Adjusted for body mass index, country of origin, and educational level.  p=0.88 for difference in association of hypertensive disorders of pregnancy and incident cardiovascular disease across categories of number of risk factors not in control (multiplicative interaction). | | | | |

| **Table S2.** Risk of incident cardiovascular disease in women with type 2 diabetes by history of hypertensive disorders of pregnancy and number of risk factors not in control | | | | |
| --- | --- | --- | --- | --- |
|  | **Crude data** (20 imputations) | | **Hazard ratios (95% CIs) from adjusted regression model**†‡ | |
|  | **Hypertensive disorders of pregnancy** | **No hypertensive disorders of pregnancy** | **Hypertensive disorders of pregnancy** | **No hypertensive disorders of pregnancy** |
| No. of risk factors not in control* | Incident rate (per 1000 person-years)† | Incident rate (per 1000 person-years)† |  |  |
| 0-1 | 4.3 | 4.0 | ref | 0.94 (0.70 – 1.26) |
| 2 | 7.1 | 6.2 | 1.56 (1.07 – 2.26) | 1.33 (0.98 – 1.79) |
| 3 | 11.4 | 9.9 | 2.41 (1.64 – 3.55) | 2.05 (1.54 – 2.74) |
| 4-5 | 18.8 | 18.0 | 3.99 (2.69 – 5.93) | 3.67 (2.69 – 5.01) |
| * Total number of the following risk factors not at target: blood pressure (≤130/80 mmHg), low-density lipoprotein levels (<2.6 mmol/l), glycated hemoglobin levels (< 53 mmol/mole), micro- or macroalbuminuria, and smoking, regardless of combination.  † Average incidence rate per strata across all imputed datasets.  ‡ Adjusted for body mass index, country of origin, and educational level.  p=0.97 for difference in association of hypertensive disorders of pregnancy and incident cardiovascular disease across categories of number of risk factors not in control (multiplicative interaction). | | | | |

| **Table S3.** Risk of incident cardiovascular disease in women with type 1 diabetes by history of hypertensive disorders of pregnancy in complete case analyses (n=3728) | | | | | | | | |
| --- | --- | --- | --- | --- | --- | --- | --- | --- |
| **Outcome** | **Hypertensive disorders of pregnancy** | | | **No hypertensive disorders of pregnancy** | | | **Regression modelling** | |
|  | Events, n | Follow-up time (person-years) | Incident rate (per 1000 person-years) | Events,n | Follow-up time (person-years) | Incident rate (per 1000 person-years) | Model | Hazard ratio (95% CI) |
| **Incident cardiovascular disease** | 28 | 6883 | 4.1 | 118 | 27,857 | 4.2 | I * | 1.15 (0.76 – 1.74) |
|  |  |  |  |  |  |  | II † | 0.90 (0.58 – 1.39) |
|  |  |  |  |  |  |  | III ‡ | 0.92 (0.60 – 1.43) |
| * Adjusted for age through age-specific model strata  † Additionally adjusted for blood pressure, low-density lipoprotein levels, glycated hemoglobin levels, presence of albuminuria, and smoking.  ‡ Additionally adjusted for body mass index, country of origin, and educational level. | | | | | | | | |

| **Table S4.** Risk of incident cardiovascular disease in women with type 2 diabetes by history of hypertensive disorders of pregnancy in complete case analyses (n=30,234) | | | | | | | | |
| --- | --- | --- | --- | --- | --- | --- | --- | --- |
| **Outcome** | **Hypertensive disorders of pregnancy** | | | **No hypertensive disorders of pregnancy** | | | **Regression modelling** | |
|  | Events, n | Follow-up time (person-years) | Incident rate (per 1000 person-years) | Events,n | Follow-up time (person-years) | Incident rate (per 1000 person-years) | Model | Hazard ratio (95% CI) |
| **Incident cardiovascular disease** | 164 | 21,412 | 7.7 | 987 | 161,214 | 6.1 | I * | 1.29 (1.09 – 1.52) |
|  |  |  |  |  |  |  | II † | 1.29 (1.09 – 1.52) |
|  |  |  |  |  |  |  | III ‡ | 1.26 (1.07 – 1.49) |
| * Adjusted for age through age-specific model strata  † Additionally adjusted for blood pressure, low-density lipoprotein levels, glycated hemoglobin levels, presence of albuminuria, and smoking.  ‡ Additionally adjusted for body mass index, country of origin, and educational level. | | | | | | | | |
